# Machine Learning Reveals a General Understanding of Printability in Formulations Based on Rheology Additives

**DOI:** 10.1002/advs.202202638

**Published:** 2022-08-25

**Authors:** Ali Nadernezhad, Jürgen Groll

**Affiliations:** ^1^ Chair for Functional Materials for Medicine and Dentistry at the Institute for Functional Materials and Biofabrication (IFB) and Bavarian Polymer Institute (BPI) University of Würzburg Pleicherwall 2 97070 Würzburg Germany

**Keywords:** bulk rheology, machine learning, printability, rheology additives

## Abstract

Hydrogel ink formulations based on rheology additives are becoming increasingly popular as they enable 3‐dimensional (3D) printing of non‐printable but biologically relevant materials. Despite the widespread use, a generalized understanding of how these hydrogel formulations become printable is still missing, mainly due to their variety and diversity. Employing an interpretable machine learning approach allows the authors to explain the process of rendering printability through bulk rheological indices, with no bias toward the composition of formulations and the type of rheology additives. Based on an extensive library of rheological data and printability scores for 180 different formulations, 13 critical rheological measures that describe the printability of hydrogel formulations, are identified. Using advanced statistical methods, it is demonstrated that even though unique criteria to predict printability on a global scale are highly unlikely, the accretive and collaborative nature of rheological measures provides a qualitative and physically interpretable guideline for designing new printable materials.

## Graphical abstract

While using formulations based on rheology additives helps to expand ink libraries for biofabrication, a lack of generalized understanding of the contributing factors hinders the development of engineered inks. Physically interpretable guidelines are provided to design new printable materials using descriptive machine learning techniques based on bulk rheological properties.

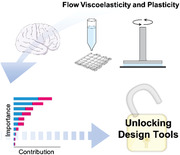

## 1 Introduction

By introducing additive manufacturing technologies to the field of tissue engineering (TE), and in particular 3D bioprinting, a significant expansion in the scope and applicability of TE approaches was achieved.^[^ ^1^ ^]^ The advancement of 3D bioprinting significantly depends on development in three critical frontiers, technological innovations,^[^ ^2^ ^]^ the discovery of new functional biomaterials,^[^ ^3^ ^]^ and deepening our understanding of regenerative biology.^[^ ^4^ ^]^ While addressing all the requirements for a successful regenerative approach might seem out of reach for the moment, significant resources have been dedicated to approximating this process. In this respect, engineering biomaterial inks and bioinks includes a relatively large portion of research in the field, exclusively exploring the enabling possibilities by introducing new synthetic and natural biomaterials and formulations.^[^ ^3^ ^]^

Apart from meeting the strict biological requirements, the materials used in the 3D bioprinting approach, or in general terms, the 3D printing of soft biomaterials, need to fulfill some physical and mechanical criteria. The primary materials used as the ink for this purpose include hydrogels or polymer solutions. Traditionally, the natural biocompatible hydrogel inks failed to meet the 3D printing prerequisites, categorized primarily by lack of printability.^[^ ^3^ ^]^ The phrase printability refers to the capability of the ink material in allowing the 3D printing process to form the designed structure with acceptable shape fidelity, mechanical stability, and structural integrity.^[^ ^5^ ^]^ Despite the extent of these measures, the printability of a hydrogel ink or polymer solution is greatly influenced by its chemistry and mechanical properties.^[^ ^3^ ^]^ Additionally, bioprinting parameters such as flow rate, printing speed, nozzle size, process temperature, and subsequent post‐printing steps could significantly influence the outcome of 3D printing.^[^ ^6^ ^]^

Due to the biological requirements, many attempts were made to enable or enhance the printability of promising known bioactive hydrogels and polymer solutions that formerly lacked physical and mechanical needs. These mainly included chemical modifications or blendings with a secondary material that could induce printability. The latter, generally referred to as formulations, is becoming increasingly popular for two main reasons. First, several highly efficient additives are already available that meet the biological requirements and can significantly enhance the printability of the base hydrogel or polymer solution. Second, and more importantly, the low cost and the straightforward know‐how of creating new formulations constitute a significant advantage over developing sophisticated chemistries to induce comparable functionalities. Although this ease of processing does not replace the offerings of an application‐tailored chemical modification, the literature shows an increasing trend in applying formulations in different domains related to the 3D printing of biomaterials.

Successful engineering of a new formulation for 3D printing needs a profound understanding of the material properties on micro and macro scales. However, in an interdisciplinary field of research such as biofabrication, there are tendencies to approximate the materials’ related requirements, mostly toward more established biological measures. In this context, the performance of an additive is mainly weighted by the corresponding biological response rather than quantification and analysis of the material's properties. Nevertheless, the available literature shows increasing awareness and willingness of research groups to design the formulations based on traditional and state‐of‐the‐art physical characterization methods.^[^ ^3^ ^]^ These mainly included the rheological characterizations of inks and finding the correlations between the printing conditions and printability of the inks.

Analysis of such systems usually requires a degree of simplification, as increasing the number of parameters and variables could quickly deteriorate the interpretability of the readout by the conventional methods. A promising tool to overcome this limitation might be the data analysis techniques employing Machine Learning (ML) principles.^[^ ^7^ ^]^ ML methods include statistical and mathematical tools which can reveal and exploit the relationships in data and deliver complex models to describe the system. Despite the visions and hopes for applying ML tools in the scope of 3D bioprinting,^[^ ^8^ ^]^ there are a few reports on using ML techniques to analyze correlations in 3D bioprinting of hydrogel inks,^[^ ^9^ ^]^ which mainly focused on providing a metric on the predictability of the printability of similar inks, based on a few materials‐ and 3D printing process‐related parameters. Moreover, process optimization is the central focus of the available literature on extrusion and droplet‐based bioprinting techniques, as the process requirements for optimization of printability in a defined material system can be approximated with reasonably low degrees of simplifications.^[^ ^9^, ^10^ ^]^ Although these studies provide a deeper insight into the dependency of printability on 3D printing process parameters by considering the performance‐processing relationship, they generally overlook the fundamental material‐related aspects due to the increased complexity of the problem.

A common approach in applied ML techniques is providing metrics and models that enable a system's predictability based on hidden correlations. By increasing the complexities of the models and their predictability power, the combinations and correlations of variables become more obscure, and interpretation of the process of making a particular decision by the model becomes inherently complicated. This problem gave rise to interpretable models, mostly recognized as explainable artificial intelligence (AI), which provide a technically equivalent but more understandable approach than black‐box models for data analysis and predictions.^[^ ^11^ ^]^

An intriguing question in the context of 3D bioprinting is whether explainable AI can describe the process of rendering printability in an unknown formulation and interpret it through measurable physical indices. To answer this question, we used bulk rheology indices (hereafter called features) in an extensive library of different formulations based on hyaluronic acid (HA) polymer solutions and three different rheology modifiers with distinct microstructural interactions. After developing a predictive ML model with high precision, we described how the knowledge from the modeled data could interpret a particular model's decision toward the classification of a formulation and demonstrate the dependency of printability on many correlations of the features.

## 2 Results

### 2.1 Production of Data with Minimized Bias

**Figure 1:**
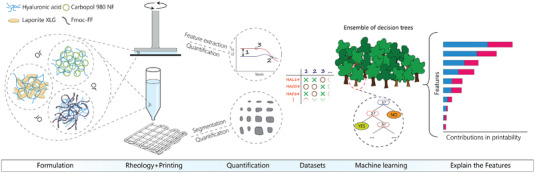
A demonstration of the multi‐steps taken in this study to explain the printability. The flow of this study is depicted from left to right of the figure. First, the formulations based on different additives and starting HA solutions were prepared. In the second step, rheological characterizations and printing experiments were performed. The quantification step involved the extraction of various rheological features and quantitative analysis of the printability of formulations. In the next step, datasets based on combining all acquired features and printability scores were fabricated. By using the generated datasets, a random forest ML algorithm was trained. In the final step, a post‐analysis of the obtained model revealed the correlations between data and the influence on making a decision by the model.

**Figure** **1** shows an overview of the multiple steps taken in this study to identify and explain the contributing factors in enabling printability by adding rheology modifiers into a not‐printable polymer solution. In the first stage, three different rheology modifiers with significantly different physical properties were selected to alter the rheology and printability of plain HA solutions with three different molecular weights at various additive ratios. In addition to the interactions between HA molecules and the additives, each rheology modifier had unique interaction mechanisms to alter the viscoelasticity of the final formulation: colloidal and granular interactions of Carbopol microgels, formation of a secondary network by electrostatic interactions between Laponite nanodiscs, and entanglement and network formation of 1‐dimensiona self‐assembled Fmoc‐FF fibrils. The information regarding the polymer: additive ratio, the additives’ type, and the initial polymer's molecular weight were not revealed to the ML algorithm at the later stages to avoid creating any bias throughout the analysis and potentially decreasing the universality of the outcome.

A multi‐step rheological testing protocol was designed to acquire information on viscoelasticity and flow properties of samples holistically. The complete list of rheological data obtained from each test step and the extracted ranges are provided in Table S1, Supporting Information. Notable, the apparent yield behavior of formulations was identified by whether a peak in the viscosity–shear stress plot was observed, and if so, the corresponding value was recorded. From the physical point of view, this peak and its value corresponds to the buildup of resistance against the flow on a macroscopic scale. The testing protocol was aimed to maximize the obtained information with minimum complexities. Especially, creep‐related tests were avoided since the preliminary experiments (data not included) for optimization of protocol showed the sensitivity of creep tests toward not‐printable formulations, potentially resulting in a bias in the analysis.

The printing experiments were conducted considering that no pure HA solution was printable. The printing experiments were performed considering the volumetric flow of each formulation during printing. A recent study by Fisch et al.^[^ ^12^ ^]^ demonstrated the sensitivity and susceptibility of pneumatic‐driven extrusion systems to over‐ or under‐extrusion if the volumetric flow and the cartesian translation feedrate are mismatched. For this reason, each formulation was printed at a unique combination of applied pressure and feedrate; the latter was derived automatically based on the extruded mass of the formulation during a given time. Two features of the printing process, the ability to form a filament and the proportionality of the volumetric flow to printing pressure, were recorded as the characteristics of the printing process per formulation.

**Figure 2:**
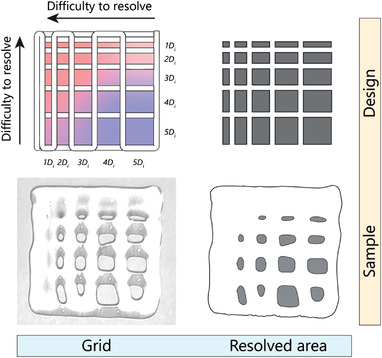
A schematic and experimental representation of the metric used in this study to quantify printability. Top‐left) a 2‐layer grid design with varying fiber spacing was used to assess the printability of the formulations. The fiber spacing was increased by increments of inner nozzle diameter (*D*_i_ = 410 µm). Bottom‐left) an image of an actual sample printed according to the grid design. Top‐right) The resolved area of design is used for benchmarking printability. Bottom‐right) The segmented image of a printed formulation is used to calculate the percentage of resolved area.

The designed path for the printability assessment included a 2‐layered rectangular mesh with a directional increase in fiber spacing based on increments of the inner diameter of the nozzle (*D*_i_) (**Figure** **2**). The 3D assessments of printability were deliberately avoided since criteria such as general 3D shape fidelity and fiber sagging supposably demand a certain extent of viscoelasticity, which could interfere with the objectives of this study in the unbiased evaluation of induced printability by rheology additives.^[^ ^13^ ^]^ Based on our preliminary screening experiments, a weighting approach to penalizing the easy‐to‐resolve areas was employed (Figure S1, Supporting Information). In general, the induced printability in different formulations was not significant, as only about 14% of the formulations could resolve more than 33% of the designed area. We speculate that several factors contributed to such behavior. Among them, the concentration restrictions (maximum concentration of additives was 2.5 wt/v% in a 1:1 additive:polymer ratio) imposed by the experimental design and potential biological requirements, and the wide range of physical properties of initial polymer solutions (resulted by variation of concentration and molecular weight) would play the critical role.

The printing parameters could significantly influence printability.^[^ ^14^ ^]^ This influence is more evident in resolving geometrical features for which an abrupt change in the printing process, such as a change in direction, is expected.^[^ ^12^ ^]^ In addition, other factors such as extrusion rate and substrate interactions potentially influence the spreading and fidelity of extruded filaments. As described earlier, the printing speed adjustment based on the volumetric flow was employed to eliminate the factor of over‐and under‐extrusion, while the same substrate was used to print all the formulations. However, to avoid complexities caused by pressure‐dependent extrusion delay, no changes in the printing speed at turning points were implemented. This phenomenon resulted in the underscoring of weakly printable formulations, as revealed by the review of the raw quantitative analysis of individual printing experiments (data not included). However, in an equally conditioned set of experiments, this could be considered a result of the lack of meeting the viscoelasticity requirements for extrusion printing.

After acquiring the rheological and printing data, the dataset was generated for further analysis by considering two feature types. Concretely, some aspects of the raw data from rheological experiments were used as the primary features. To include the additive nature of the rheology modifiers in the analysis, most of the extracted primary features of formulations were individually divided by the corresponding values of the respective HA solutions of different concentrations and molecular weights to include the proportion of change in further analysis.

The second type included combinatorial features. The combinatorial features were generated based on combining some aspects of the data collected during experiments, and as a principle, all the primary and combinatorial features were selected based on physically explainable factors rather than abstract quantities. The details of the complete list of features are provided in Table S2, Supporting Information.

### 2.2 Selection of Relevant Features Influencing Printability

**Scheme 1:**
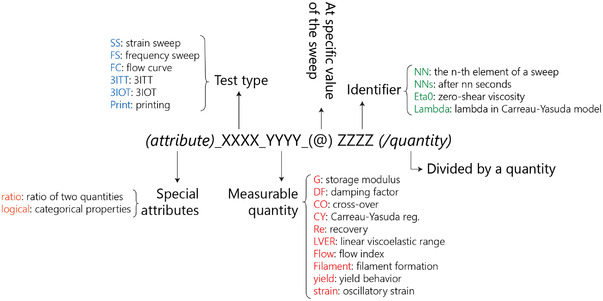
Reference for the nomenclature used to address different features in this study.

The data from rheological and printability experiments of all the formulations were prepared and consolidated into a single structured data frame. An intuitive nomenclature system was devised to identify the different features in the dataset. The complete list of nomenclature used to address different elements in the dataset is provided in Table S2, Supporting Information. To increase the legibility of the manuscript, **Scheme** **1** provides the guideline for interpreting the coded nomenclatures.

Spearman's correlation analysis of the raw data showed significant dependencies between features (Figure S2, Supporting Information). By implementing the statistically robust algorithm of Boruta feature selection,^[^ ^15^ ^]^ the original features were refined to those that significantly contributed to printability prediction with a high F‐score of a trained random forest (RF) model. Boruta algorithm identifies the importance of the features for constructing the ML model based on the performance of a randomized version of the features through many iterations. Eventually, the statistically impactful features are selected from the top 0.5% of the binomial distribution of the iterations. This procedure resulted that, among the initial 65 features, only 13 were identified as having a significant influence on the predictability of the RF model.

The performance of the RF model for feature selection and on the refined dataset was assessed using F‐score through *n*‐repeated *k*‐fold cross‐validation (*n* = 20, *k* = 5). The RF model consisted of 200 estimators using bootstrap aggregation. Moreover, the out‐of‐bag (OoB) error was evaluated to estimate the performance of the bagged model. **Table** **1** lists the performance of the RF model for feature selection by the Bruta algorithm on the full‐feature dataset and the performance metrics of the RF model on the refined dataset.

**Table 1:** Performance metrics of the RF model for feature selection on the full‐feature dataset and after refining the dataset

—	—	Average F‐score	Min (F1‐score)	Max (F1‐score)	StDev (F1‐score)	Average OoB‐score
Full dataset	Model^a)^	—	—	—	—	0.95
—	Printable	0.91	0.83	1.00	0.058	—
Refined dataset	Model^b)^	—	—	—	—	0.96
—	Printable	0.94	0.91	1.00	0.039	—
—	Not‐Printable	0.99	0.98	1.00	0.007	—

^a)^ Data from 10 000 iterations on (*n*) repeated *k*‐fold cross‐validation (*n* = 20, *k* = 5) on full‐feature dataset.

^b)^ Data from (*n*) repeated *k*‐fold cross‐validation (*n* = 20, *k* = 5)

**Figure 3:**
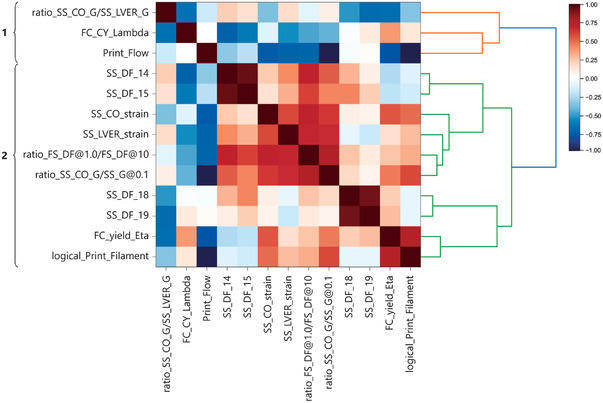
The dendrogram shows the Spearman rank‐order correlation matrix and the linkage according to hierarchical cluster analysis of Spearman's correlations. The numbers on the left identified two clusters. A value of 1 or −1 in the correlation matrix denotes high correlations, and the direction of association depends on the sign of the correlation coefficient.

To explore and understand the existing correlations between these essential features, the Spearman rank‐order correlation matrix and the linkage based on hierarchical cluster analysis of Spearman's correlations are demonstrated in **Figure** **3**. This analysis showed two main clusters of features (as indicated by different colors of the dendrogram) with distinct linkage distances from each other.

These two clusters include features that could be physically interpreted as 1) describing the ease of creating flow and its plasticity (orange leaves in Figure 3) and 2) describing the homogeneity of the flow and the viscoelasticity of the formulation before and after the flow (green leaves in Figure 3).

The first cluster involves the ratio between storage modulus at the flow point and the limit of the linear viscoelastic range, the consistency index determined by the Carreau–Yasuda model, and the proportionality index of the flow during printing. The first feature describes the plasticity of the formulation before the flow, while the consistency index indicates the necessary shear rate for the transition from Newtonian to non‐Newtonian flow regime. The flow proportionality index reflects the requirements for maintaining the formulation's flow during the extrusion through a fine nozzle. The information from these three features complements each other, describing correlations between the ease of creating the flow in a formulation and the force requirements to maintain the flow during the extrusion.

The second cluster includes two primary nodes, one describing the viscoelasticity of the formulations, and the other includes features related to the resistance against initiating the flow and the extent of viscous deformation afterward. On the one hand, the viscoelasticity of the formulation prior to flow was critical in predicting printability. The dominancy of the viscous portion of deformation prior to flow, determined by the damping factors at moderate shear strains (14.8% and 21.7%), was correlated with the required strain to initiate the flow, the extent of linear viscoelasticity, the extent of stability of the interactions by changing the frequency of deformation, and the energy required to induce the flow of formulation with consideration of the elasticity of the starting HA solution (ratio between storage modulus at low strain and flow point multiplied by the storage modulus of corresponding HA at 0.1% strain).

On the other hand, the closely correlated damping factors at high shear strains (68.5% and 100%) highlight the contributions of the viscous nature of the formulation in its printability, which is linked to the likelihood of filament formation during the extrusion and the yield viscosity of the formulations.

Although the correlative analysis showed the contribution of different rheological characteristics of the formulations in creating printability, their extent is yet to be determined.

### 2.3 Global and Local Explanation of Printability

Despite identifying the important features that contributed to printability prediction, the extent of the contributions is unknown. This is a typical characteristic of models generated with most ML methods, as interpreting the predictive model's output is tedious, especially by increasing the complexities in non‐linear models. Shapley additive explanations (SHAP) is a powerful tool for interpreting the prediction by ML models.^[^ ^11c^ ^]^ SHAP has a solid theoretical foundation in game theory and can provide contrastive explanations and analyze the model's output locally and globally.

**Figure 4:**
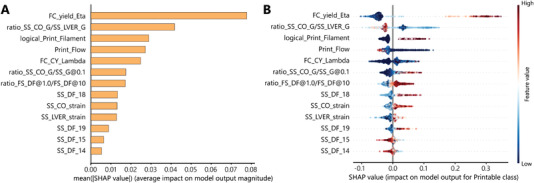
SHAP values of different features show their contributions to the model output on two scales. A) on a global scale, the mean SHAP value represents the feature's average impact on the predictions made by the model. B) on a local scale, the rank‐ordered features explain the margin output of the model, which is the change in printability of formulations. The plot also shows the range of influence over the dataset. The color shows how the change in the value of a feature affects the change in the printability prediction.

The extent of the contribution of different features in the predicted printability of formulations is demonstrated in **Figure** **4**A. The average SHAP value quantifies the impact of each feature on the model output by interpreting the average expected contribution of the feature after all the possible combinations of other features are considered.

The SHAP feature importance plot shows that the most important features in the global scale for printability prediction included the yield viscosity, and the ratio of storage modulus at the flow point to that of the limit of the viscoelastic range (plasticity of the formulation before flow). Although the impact of the other features on the model's output is to a lesser degree, their contributions to the model's accuracy cannot be disregarded since the SHAP analysis was performed on all the relevant features selected by the Boruta algorithm.

The average SHAP value provides information about the contribution of the features on a global scale. However, on a local scale, evaluation of individual observations and the corresponding SHAP value demonstrates how each feature contributed to predicting a printable formulation experimentally (Figure 4B). Careful analysis of Figure 4B indicates that different features contributed in different directions to enabling printability.

For instance, a high yield viscosity of the formulation resulted in a higher printability score, while lower plasticity prior to the flow contributed to more printable formulations. The lack of filament formation ability negatively influenced the printability of the formulations. Additionally, formulations with a lower flow proportionality index were statistically more susceptible to be identified as printable, meaning that either a lesser extrudate mass at constant pressure or larger force to extrude the same mass of the ink determines a printable formulation. This feature directly corresponds to the microstructural interactions of the formulation; the stronger the interaction, the better resistance to deformation and better printability. Moreover, moderate to high values of the consistency index in the Carreau–Yasuda model correspond to printable formulations, translating to transition to a shear thinning behavior at moderate to low shear rates.

A higher ratio of storage modulus at the flow point to the one at 0.1% strain (proportional to the starting HA's storage modulus) resulted in a higher probability of printability, meaning that the higher elasticity of starting HA solution could contribute to more printability of the formulation. Similarly, a higher ratio between the damping factor at low and high deformation frequencies contributed more to the printability of the formulation. From the physical point of view, this meant that lesser variation of the damping factor in a range of frequencies results in a higher chance of printability. In other words, more stability of the interactions at the moderate to the low portion of the frequency spectrum results in better printability.

More pronounced viscous behavior after the flow (at shear strains of 68.5% and 100%) contributed more to the printability of formulations. Moreover, the higher flow strain also resulted in better printability of the formulations, and similarly, formulations with moderate to high levels of the limit of linear viscoelastic range showed more printability. Finally, the dominant elastic behavior at lower shear strains (14.8% and 21.7%) contributed positively to printability, indicating that the dominancy of elastic behavior at low strain values positively influences the formulations’ printability.

The outcome of SHAP feature importance in a complex model is inevitably context‐dependent, meaning that a contrastive SHAP statement might not hold for all experimental conditions. The dependency of the model's outcome on every single feature, and eventually on their combinations, can show the relationship between the features and the target. In order to clarify the validity of the SHAP analysis, we performed the partial dependence (PD) analysis to show the average marginal effects of features on the outcome of the printability assessment (Figures S3 and S4, Supporting Information). Although the assumption of independence for highly correlated features (such as damping factors at low or high strain values) is not necessarily valid, however, the outcome of PD analysis showed that with some degrees of simplifications, the extent of dependency of the output on the features is likely to increase by increasing the feature importance determined by SHAP.

**Figure 5:**
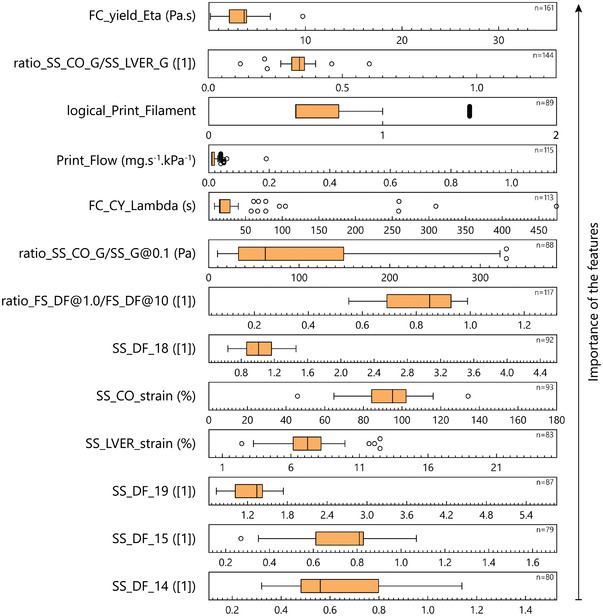
Decision rules in the random forest algorithm are shown by box‐and‐whisker plots (Tukey, outliers in open circles) across the full range of each feature. The importance of the features increased from the bottom to the top of the graph. The number of total splitting nodes based on each feature is indicated by *n*.

Evaluation of decision rules for every decision tree of the random forest algorithm revealed that by increasing the importance of a feature, a narrower range for the feature's threshold value could be identified (**Figure** **5**). As a result, only a few features could be used to determine relatively more precise boundaries for distinguishing a printable formulation from a non‐printable one. In contrast, the major part of the features contributed to determining printability across a wide range of values. This was a significant finding, as it hints toward the collaborative role of rheological characteristics of a formulation in rendering printability, rather than introducing a dominant single measure to classify formulations.

**Figure 6:**
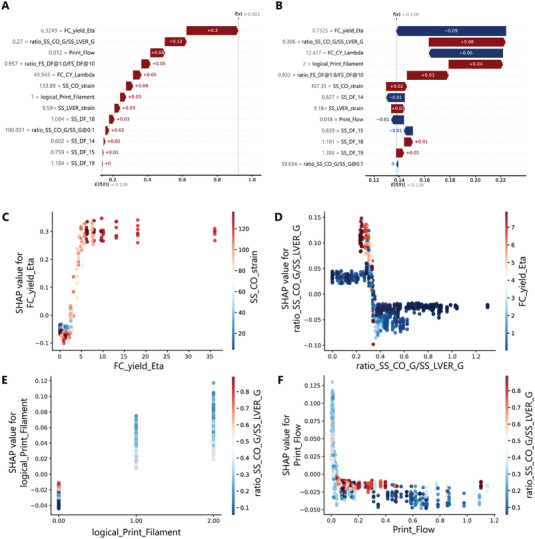
The explanations for individual predictions and SHAP dependence plots of some notable combinations of features. The Waterfall plots showing the collaborative influence (positive: red, negative: blue) of rheological characteristics in a randomly chosen prediction resulted in A) significantly positive offset of the model's output (dashed vertical lines) from the baseline value of the model (*E*(*f*(x)), and B) an accumulative contribution resulting in a negligible offset of the model's output from the baseline value. Grey numbers indicate the actual value of the features per observation. The dependency plots show the SHAP value for C) yield viscosity, D) plasticity of formulation before the flow, E) the likelihood of filament formation during the extrusion, and F) the proportionality index of the flow.

The collaborative contributions of a formulation's rheological characteristics toward printability are better demonstrated within the observations made by the algorithm (**Figure** **6**A,B). Figure 6A shows the contributions of all the rheological features in a randomly picked observation by the model, which was later identified as a printable formulation. As expected, the most impactful features contributed significantly toward printability. However, the minor contributions of the other rheological characteristics are critical, as also demonstrated in a scenario where the more important model features have a less dominant or negative influence on the output based on their values (Figure 6B). In such cases, certain contributions of the features might be canceled out with the others, eventually leading to a decision based on mixed contributions of the rheological characteristics to the model.

In addition to the individual contributions, a significant amount of mutual impacts from different factors could occur in a multi‐variable system. Figure 6C–F shows four of the most notable dependencies observed between the features of the models. Every dot in plots of Figure 6C–F corresponds to one prediction, and a SHAP value above zero meant a positive contribution of the feature in that prediction toward being classified as a printable formulation. In contrast, a value below zero negatively influenced the prediction outcome. It should be noted that a positive SHAP value for each feature in these dependency plots does not mean that predicting a formulation as printable is guaranteed. Instead, it shows that a particular feature's positive contribution could depend on another feature's value. In this way, the higher strain required to create the flow resulted in a higher SHAP value for the yield viscosity, or a higher SHAP value for the degree of plasticity was observed when the formulations with lower plasticity before the flow had a higher yield viscosity (Figure 6C,D). While the formulations with no filament formation during the extrusion had negative SHAP values regardless of the degree of plasticity of the formulation, the lower degree of plasticity resulted in positive SHAP values for the likelihood of filament formation during the extrusion in case one was formed (Figure 6E). Moreover, a dependency between the flow's consistency index and the degree of plasticity was observed. A moderate degree of plasticity at low consistency indices resulted in a more prominent contribution of the flow index to printability, as reflected by the higher SHAP value for the flow's consistency index. (Figure 6F).

## 3 Discussion

Current literature on assessing, evaluating, and predicting the printability of soft inks and bioinks mainly relies on case studies or the correlation between 3D extrusion‐based printing process parameters.^[^ ^5^, ^14^, ^16^ ^]^ Several studies focused on optimizing the 3D bioprinting process in terms of printability and cell viability in extrusion and droplet‐based techniques.^[^ ^6^ ^]^ Printability of a formulation is a result of the complex interactions between different physical properties of additives and the base inks, together with the requirements of the process. The formulations investigated in this study involved a range of characteristic behaviors and included three rheology additives with essentially different physics for inducing printability. From a general point of view, the mechanisms driving the reinforcement and variation of viscoelasticity in these formulations could vary. Additionally, some factors such as chemistry and affinity of the additives and base polymer solutions could significantly change the mechanisms and outcome of interactions. These fundamental aspects were not separately considered in this study. Nevertheless, the rheological features investigated in this study reflected the formulations’ material‐dependent properties.

Similar to some of the findings of this study, it has been shown previously that in a colloidal system, critical flow‐related and viscoelastic indices such as yield stress, stiffness and plasticity of the system, flow strain, and the flow transition index exhibited some patterns and distinct behaviors, which could hint toward a printable formulation.^[^ ^17^ ^]^ However, such criteria's universality and applicability to different material systems have not been further studied.

The ML model identifies printable formulations in a pool of many observations based on knowledge of correlations and interactions during training. Apart from metrics to describe the model's accuracy, the conditions that resulted in recognizing the printable formulations with high accuracy are the key subjects in explaining how a new formulation with a given set of attributes would be classified. Analogically, the rationale behind making a particular model prediction is comparable to tuningsome principal and dependant physical properties of the base polymer solution by adding additives that would enhance or worsen printability from an experimental point of view.

The few available studies on the application of ML in the field of 3D (bio)printing focused on either investigating a group of inks with similar characteristics or a set of process parameters with minimum alterations in ink properties.^[^ ^9^ ^]^ The rationale behind limiting the factors is very reasonable, considering the interpretability of the outcome and the required resources. However, we showed that it is possible to generate a physically interpretable model by diversifying the training pool to include several types of formulations. The quantification of the decision rules revealed that only a few features showed a distinct threshold that might be used as metrics in the rough data screening. Beyond that, it is highly unlikely to accurately describe printability by disregarding the rest of the rheological features, as they showed a collaborative influence on the overall printability induced in the formulation.

For the first time in the literature, we showed a generalized correlation between different rheological factors describing the printability of a hydrogel ink formulation. Accordingly, the obtained model predicts that from a statistical point of view, a formulation becomes printable when it shows a high yield viscosity and a low degree of plasticity before the flow, while the transition from Newtonian to non‐Newtonian behavior of the flow occurs at relatively low shear rates. The formulation tends to flow at higher pressures during printing, and extruding through a small nozzle forms a filament rather than a droplet. The formulation based on polymer solutions with higher elasticity tends to be more printable, and a higher degree of stability of microstructural interaction over a range of frequencies is favored. While generally, a more extended range of linear viscoelasticity is desired, the damping factor of the formulations at low and high strain values should follow a pattern, as a formulation with more elasticity at the lower range and high viscous nature at the higher range is more desired.

## 4 Experimental Section

### Materials

Hyaluronic acid sodium salt with three different molecular weights was purchased from Carbosynth (*M*_w_ 0.6–1.0, 1.0–2.0, and 2.0–2.5 MDa; Biosynth Carbosynth, Compton, UK). Carbopol 980 NF was purchased from Lubrizol (Lubrizol Pharmaceuticals, OH, USA). Laponite XLG was purchased from BYK Additives (BYK‐Chemie GmbH, Germany). *N*‐fluorenylmethoxycarbonyl diphenylalanine (Fmoc‐FF) was purchased from Bachem (Bachem, Switzerland). Dimethylsulfoxide (DMSO) was purchased from Sigma‐Aldrich (Sigma‐Aldrich, USA). Sodium hydroxide was purchased from Merck (Merck KGaA, Germany).

### Formulations

HA‐Carbopol (HAC), HA‐Lapointe XLG (HAL), and HA‐Fmoc‐FF (HAF) formulations were prepared with different concentrations and starting HA molecular weights by thoroughly mixing the required amounts of HA and the respective additive's stock solutions as listed in **Table** **2**. All the formulations were incubated at 4 °C for 24 h after mixing. 1 h of equilibrium time at room temperature was administered before each measurement.

**Table 2:** Different formulations based on additives used in this study

Formulation	HA: Additive	Total concentration [mg mL^−1^]	HA Mw [MDa]
HAC	10:10 to 10:01	15	0.6–1.0
—	—	—	1.0–2.0
—	—	—	2.0–2.5
—	—	30	0.6–1.0
—	—	—	1.0–2.0
—	—	—	2.0–2.5
HAL	10:10 to 10:01	15	0.6–1.0
—	—	—	1.0–2.0
—	—	—	2.0–2.5
—	—	30	0.6–1.0
—	—	—	1.0–2.0
—	—	—	2.0–2.5
—	—	45	1.0–2.0
—	—	—	2.0–2.5
—	—	50	0.6–1.0
HAF	10:10 to 10:01	5	0.6–1.0
—	—	—	1.0–2.0
—	—	—	2.0–2.5

For HAC formulations, the stock solution of Carbopole at 30 mg mL^−1^ was prepared in MilliQ water, and the pH of the solution was neutralized with the dropwise addition of 10n sodium hydroxide solution. The 100 mg mL^−1^ stock solution of Fmoc‐FF in anhydrous DMSO was used to prepare HAF formulations. The Fmoc‐FF stock solutions were prepared freshly. To prepare HAL formulations, 55 mg mL^−1^ stock solutions of Laponite‐XLG in MilliQ water were prepared. Stock solutions of HA with different concentrations and molecular weights in MilliQ water were prepared by vigorous shaking at 250 rpm at 40 °C overnight, using New Brunswick Innova 40 incubator shaker (Eppendorf, Germany).

### Rheology

An Anton Paar MCR702 rheometer with a 25 mm parallel plate geometry at a 500 µm gap was used to analyze the formulations. A general protocol for rheological measurements was designed and followed for each experiment. The protocol included the following steps: 1) Homogenizing the sample by constant rotation at 1.0 s^−1^ for 60 s. 2) Frequency sweep between 0.1–100 rad s^−1^ at 0.1% strain. 3) Amplitude sweep at 10 rad s^−1^ in logarithmic scale between 0.01–500% strain. 4) 3‐Interval Thixotropy Test (3ITT) at 1.0, 100, and 1.0 s^−1^ shear rates. The recovery viscosity was calculated as the percentage of the rest viscosity at 5, 10, and 30 s. 5) 3‐Interval Oscillatory Test (3IOT) at 10 rad s^−1^ with 0.5%, 50%, and 0.5% strain. The recovery storage modulus was calculated as the percentage of the rest storage modulus at 5, 10, and 30 s. 6) Shear stress sweep in rotation in linear scale from 1.0 to 100 Pa with 0.5 Pa increments. 7) Transient shear steps with shear rates in logarithmic scale from 0.1 to 100 s^−1^, using a dynamic data acquisition method. The viscosity at each discrete shear rate value was monitored every 100 ms, and the corresponding viscosity value was reported if a 0.5% tolerance threshold over ten observed values was met. Viscosity versus shear rate curve was generated using the acquired data, followed by fitting with the Carreau–Yasuda model.

A rest period between each measurement step was implemented to recover the sample after deformation. Samples were gently loaded from standard 5 mL syringes on the lower plate without any needles attached for each experiment.

In order to benchmark the change in rheological properties induced by the additives, the equivalent HA solution of each formulation with the relevant concentration and molecular weight was tested with the same general protocol.

### Printability Assessment

The printability of each formulation was quantified based on the ability to resolve 2‐layered mesh patterns with varying inter‐fiber distances (Figure 2). Printing was performed using the RegenHU Discovery bioprinter (RegenHU, Switzerland). The hydrogels were loaded in 3 mL Luer lock syringe barrels (Nordson EFD, USA), equipped with a blunt G22 general‐purpose dispensing tip (inner diameter 410 µm, Nordson EFD, USA). For each formulation, the minimum air pressure of the pneumatic dispensing unit, which resulted in constant flow, was used. The path plans and the printing speed for each formulation were created using an in‐house developed VisualBasic program created in VisualStudio (Microsoft, USA). The layer height for printing was set to 2/3 of the nozzle diameter to ensure sufficient contact during printing. The printing speed for each sample was automatically calculated based on the extruded mass during 20 s of extrusion with the set pressure. The images of three printed grids were acquired 3 min after printing and were further quantified using Fiji.^[^ ^18^ ^]^ The printability index was calculated as the ratio between the resolved and expected area of the grid, weighed by the difficulty index of resolving a specific mesh area (Figure S1, Supporting Information). Larger grid areas of the designed path plan were penalized by a lower weight. A ratio above 0.33 was considered printable.

### Machine Learning Algorithm: Data Generation

Rheological data and printing conditions related to each formulation were processed according to the template provided in Table S1, Supporting Information. To avoid skewness of the dataset due to possible measurement errors at high and low ends of the frequency and strain sweeps, a clipped range of data was used by limiting the angular frequency and oscillatory strain values between 1.0–10 rad s^−1^ and 0.1–100%, respectively. Using a MATLAB script, the generated tabulated data were consolidated into a randomly distributed dataset with 65 features per formulation (MathWorks, USA).

### Machine Learning Algorithm: ML Model and Selection and Evaluation of Relevant Features

A classification ML model based on an RF algorithm was implemented in Python using the scikit‐learn package.^[^ ^19^ ^]^ The RF classifier is an ensemble non‐parametric model based on many decision trees. In order to build an ML model including the features with the relevant and statistically meaningful contributions, a Python implementation of the Boruta all‐relevant feature selection method^[^ ^15^ ^]^ was used (BorutaPy). No data was rejected during training to compensate for the dataset imbalance (positive sample population ≈14%). Instead, iterative randomization steps were employed to compensate for the imbalance in the dataset. Initially, a subset of the dataset with a balance of 75:25 for not‐Printable:Printable classes was randomly chosen from the original dataset. An RF classifier was trained with the subset, and if the F‐score (Equation (1)) of the trained model on the test portion of the subset was above 0.80, a Boruta feature screening was subsequently applied. This process was iterated 10 000 times, and the most‐occurring relevant features were selected from the accumulated responses of the Boruta feature screening algorithm. The F‐score was calculated by the following equation:

(1) $$F-score=\frac{TP}{TP+\frac{1}{2}\left(FP+FN\right)}$$

where *TP*, *FP*, and *FN* are the classifier's true‐positive, false‐positive, and false‐negative predictions, respectively.

Shapley values were used as the principal way of describing features’ contributions in the implemented ML model. For this purpose, SHAP was employed to explain features’ correlations, interactions, and contributions to predictions.^[^ ^20^ ^]^ SHAP values were obtained and accumulated through *n*‐repeated *k*‐fold cross‐validation of the trained ML model on random splits of the dataset (*n* = 20, *k* = 5).

## Conflict of Interest

The authors declare no conflict of interest.

## Supporting information

Supporting Information

Supporting Information

## Data Availability

The data that support the findings of this study are available from the corresponding author upon reasonable request.
