# Supplementary material for: Machine Learning Reveals a General Understanding of Printability in Formulations Based on Rheology Additives
Source: Adv Sci (Weinh). 2022 Aug 25;9(29):2202638. doi: 10.1002/advs.202202638 (PMC9561784; doi:10.1002/advs.202202638)
Supplement: Supplementary file 1 — Supporting Information [file ADVS-9-2202638-s002.pdf]

ADVANCED SCIENCE
Open Access

Supporting Information

for *Adv. Sci.*, DOI 10.1002/advs.202202638

Machine Learning Reveals a General Understanding of Printability in Formulations Based on Rheology Additives

Ali Nadernezhad and Jürgen Groll**

Supporting Information

Machine learning reveals a general understanding of printability in formulations based on rheology additives

Ali Nadernezhad and Jürgen Groll**

Chair for Functional Materials for Medicine and Dentistry at the Institute for Functional Materials and Biofabrication (IFB) and Bavarian Polymer Institute (BPI), University of Würzburg, Pleicherwall 2, 97070 Würzburg, Germany

* Correspondence to: ali.nadernezhad@fmz.uni-wuerzburg.de; juergen.groll@fmz.uni-wuerzburg.de

Template for collection of features from rheology experiments

The rheological data from the testing protocol were collected according to Table S1. The data obtained from rheological experiments were further processed in later steps to generate features.

Table S1. List of rheological data used for further processing and generation of features

Test type	Measured quantities	The range used for further processing
Frequency sweep	Storage modulus (G)	Angular frequency: 1.0-10 rad/s
	Damping factor (DF)	Angular frequency: 1.0-10 rad/s
Amplitude sweep	Storage modulus (G)	Oscillatory strain: 0.1-100%
	Damping factor (DF)	Oscillatory strain: 0.1-100%
3-Interval Thixotropy Test (3ITT)	The percentage recovery of viscosity during the third interval	Time: 5, 10, and 30 seconds
3-Interval Oscillatory Test (TIOT)	The percentage recovery of storage modulus during the third interval	Time: 5, 10, and 30 seconds
Shear stress sweep	Viscosity	Rotational shear stress: 1-100 Pa
Transient shear steps	Viscosity	Shear rate steps: 0.1-100 s^{-1}

List of features used for training the ML model

Table S2 lists all the features used to train the ML model in this study. The description of each coded feature is provided for a better understanding of each feature.

Table S2. List of full features and their corresponding descriptions

Feature	Description	Proportioned[1]
logical_FS_CO	If a crossover in frequency sweep was observed	NA
FS_DF_6	Damping factor at 1.0 rad/s angular frequency	Yes
FS_DF_5	Damping factor at 1.58 rad/s angular frequency	Yes
FS_DF_4	Damping factor at 2.51 rad/s angular frequency	Yes
FS_DF_3	Damping factor at 3.98 rad/s angular frequency	Yes
FS_DF_2	Damping factor at 6.31 rad/s angular frequency	Yes
FS_DF_1	Damping factor at 10.0 rad/s angular frequency	Yes
ratio_FS_DF@1.0/FS_DF@10	Proportioned ratio between Damping factor at 1.0 rad/s to DF at 10.0 rad/s	NA
logical_SS_CO	If a crossover in Amplitude sweep was observed	NA
SS_G_1	Storage modulus at 0.1% strain	Yes

SS_G_2	Storage modulus at 0.148% strain	Yes
SS_G_3	Storage modulus at 0.217% strain	Yes
SS_G_4	Storage modulus at 0.318% strain	Yes
SS_G_5	Storage modulus at 0.467% strain	Yes
SS_G_6	Storage modulus at 0.685% strain	Yes
SS_G_7	Storage modulus at 1.0% strain	Yes
SS_G_8	Storage modulus at 1.48% strain	Yes
SS_G_9	Storage modulus at 2.17% strain	Yes
SS_G_10	Storage modulus at 3.18% strain	Yes
SS_G_11	Storage modulus at 4.67% strain	Yes
SS_G_12	Storage modulus at 6.85% strain	Yes
SS_G_13	Storage modulus at 10.0% strain	Yes
SS_G_14	Storage modulus at 14.8% strain	Yes
SS_G_15	Storage modulus at 21.7% strain	Yes
SS_G_16	Storage modulus at 31.8% strain	Yes
SS_G_17	Storage modulus at 46.6% strain	Yes
SS_G_18	Storage modulus at 68.5% strain	Yes
SS_G_19	Storage modulus at 100.0% strain	Yes
SS_DF_1	Damping factor at 0.1% strain	Yes
SS_DF_2	Damping factor at 0.148% strain	Yes
SS_DF_3	Damping factor at 0.217% strain	Yes
SS_DF_4	Damping factor at 0.318% strain	Yes
SS_DF_5	Damping factor at 0.467% strain	Yes
SS_DF_6	Damping factor at 0.685% strain	Yes
SS_DF_7	Damping factor at 1.0% strain	Yes
SS_DF_8	Damping factor at 1.48% strain	Yes
SS_DF_9	Damping factor at 2.17% strain	Yes
SS_DF_10	Damping factor at 3.18% strain	Yes
SS_DF_11	Damping factor at 4.67% strain	Yes
SS_DF_12	Damping factor at 6.85% strain	Yes
SS_DF_13	Damping factor at 10.0% strain	Yes
SS_DF_14	Damping factor at 14.8% strain	Yes
SS_DF_15	Damping factor at 21.7% strain	Yes
SS_DF_16	Damping factor at 31.8% strain	Yes
SS_DF_17	Damping factor at 46.6% strain	Yes
SS_DF_18	Damping factor at 68.5% strain	Yes
SS_DF_19	Damping factor at 100.0% strain	Yes
SS_LVER_strain	Strain at the limit of linear viscoelastic range	No
SS_LVER_G	Sorage modulus at the limit of viscoelastic range	Yes

SS_CO_strain	Crossover strain in Amplitude sweep	No
ratio_SS_CO_strain/SS_LVER_strain	Ratio between crossover strain and the strain at the limit of viscoelastic	NA
ratio_SS_CO_G/SS_G@0.1	Ratio between storage modulus at crossover and proportioned storage modulus at 0.1% strain	NA
ratio_SS_CO_G/SS_LVER_G	Proportioned ratio between storage modulus at crossover and storage modulus at the limit of the viscoelastic range	NA
logical_FC_yield	If a peak viscosity in the stress sweep test was observed	NA
FC_yield_Eta	Peak viscosity in the stress sweep test	No
FC_CY_Eta0	Zero-shear viscosity from Carreau-Yasuda model	Yes
FC_CY_Lambda	Consistency index (λ) from Carreau-Yasuda model	No
3ITT_Re_5s	Recovery of viscosity in 3ITT after 5s	Yes
3ITT_Re_10s	Recovery of viscosity in 3ITT after 10s	Yes
3ITT_Re_30s	Recovery of viscosity in 3ITT after 30s	Yes
3IOT_Re_5s	Recovery of storage modulus in 3IOT after 5s	Yes
3IOT_Re_10s	Recovery of storage modulus in 3IOT after 10s	Yes
3IOT_Re_30s	Recovery of storage modulus in 3IOT after 30s	Yes
Print_Flow	Proportionality index of extruded volume to the applied pressure	No
logical_Print_Filament	If a filament was formed during extrusion (no filament, broken filament, yes)	NA

[1] Proportioned ratio was calculated by dividing the respected value by that of the corresponding HA solution with the same molecular weight and concentration (Yes, No, NA=Not Applicable)

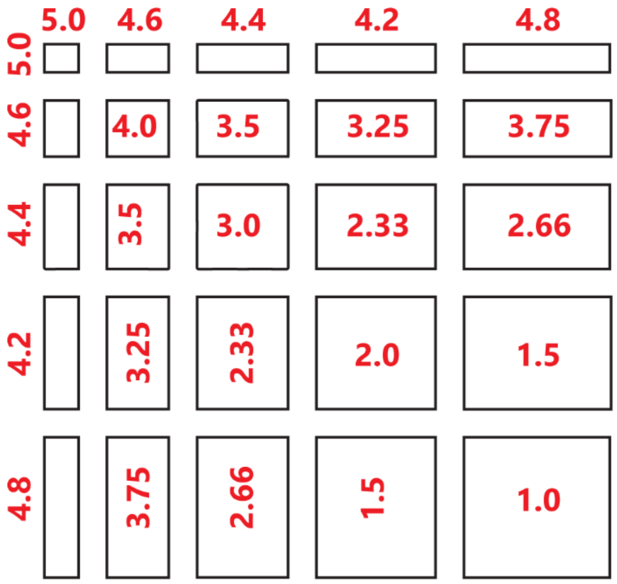

Figure S1. The weighting approach was used to penalize the easy-to-resolve areas of the grid. The numbers next to or inside each grid show the multiplication factor.

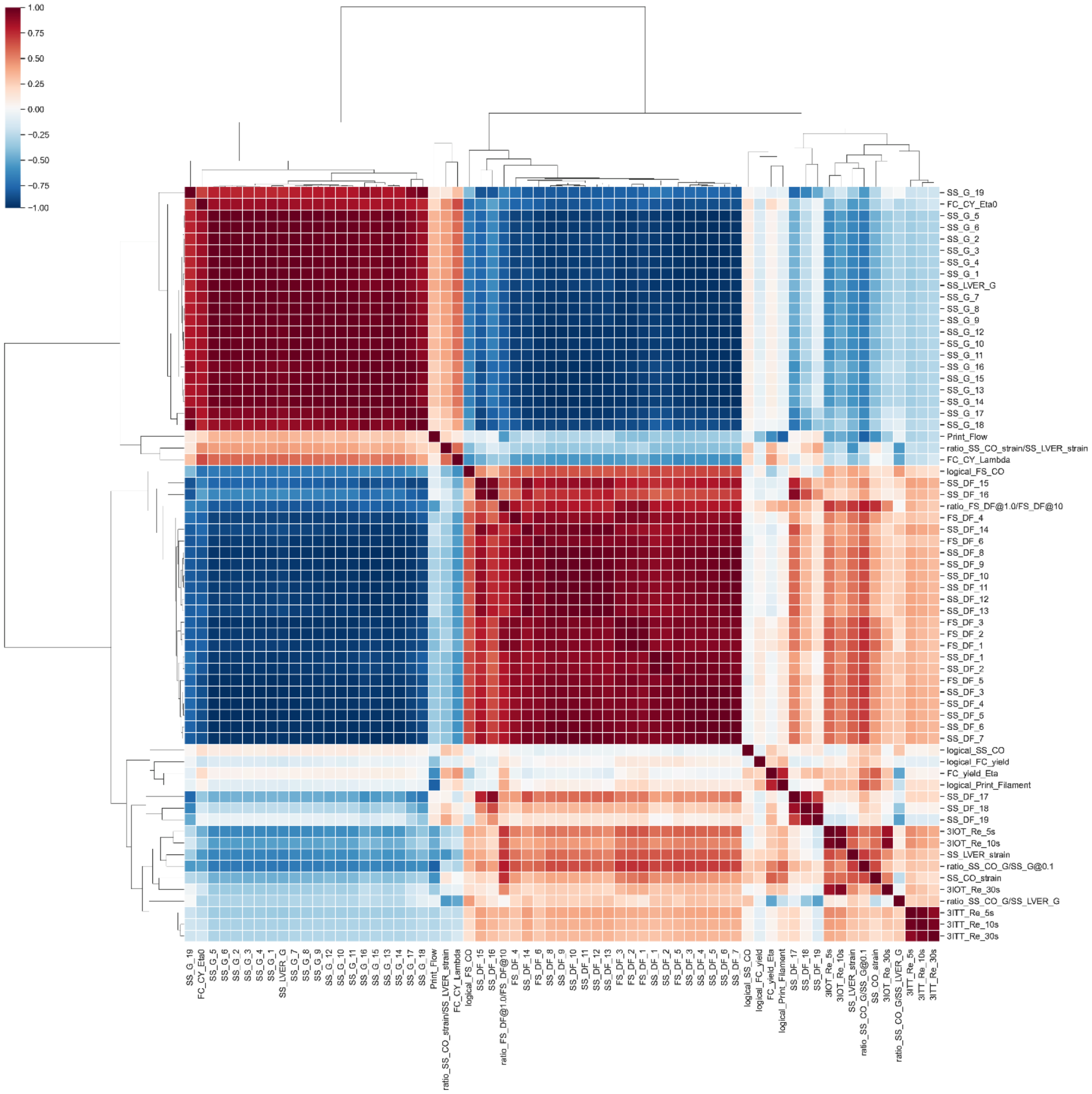

Figure S2. The results of Spearman's correlation analysis. A value of 1 or -1 denotes high correlation, and the direction of association depends on the sign of the correlation coefficient.

Dependency of the predictions made by the RF model on features

The Partial Dependence Plots (PDPs) in **Figure S3** and **Figure S4** show the average marginal effect of each feature on the predicted outcome. This analysis showed a monotonic relationship between the response and the most important features determined by SHAP analysis (Figure S3). Moreover, the two-way PDPs in Figure S3D-F show the dependence of the printability on the mutual influence of the first three impactful features, as determined by SHAP.

The model's predictive performance depended on the features' value; the impact varied depending on the features' importance. In this way, the most impactful features (as determined by SHAP) in Figure S3 had a considerably significant influence on the predicted printability, while this influence was observed to a lesser degree by decreasing the feature importance (Figure S4).

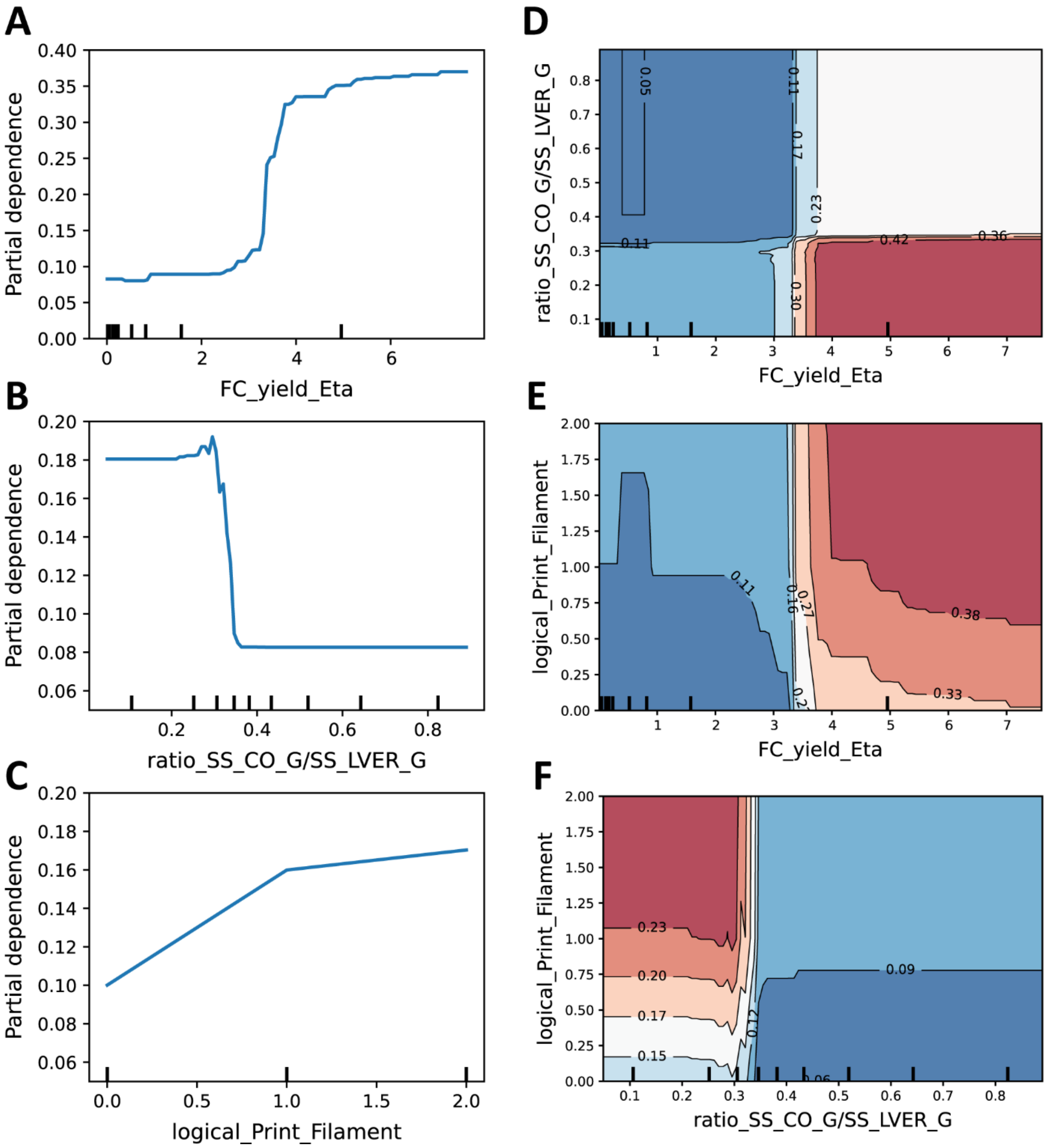

Figure S3. Partial dependence plots of the three most important features on the predicted printability. A-C) dependence of the predicted printability on each feature's value. D-F) the two-way PDPs showing the dependence of predicted printability on the joint values of three important features.

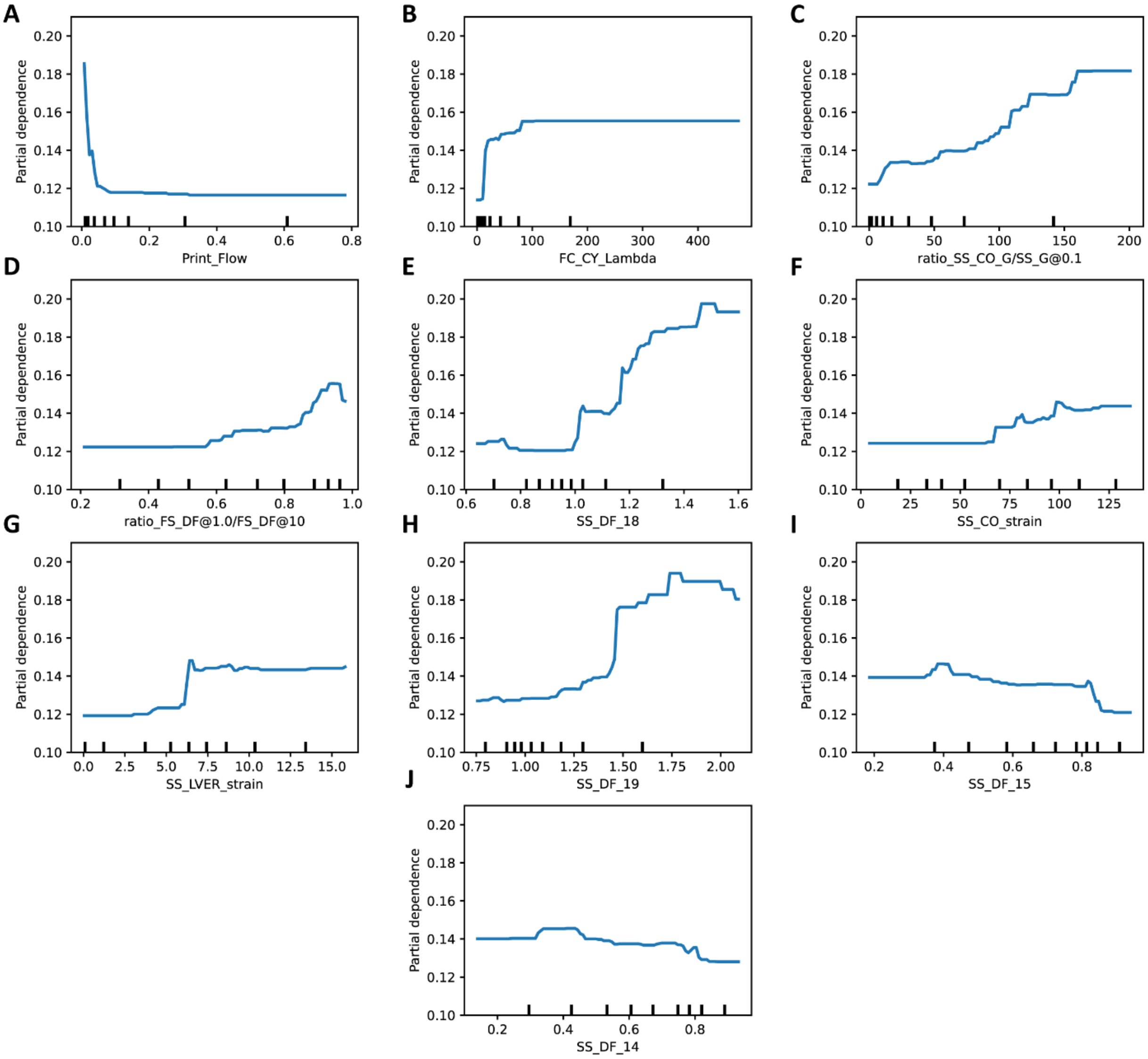

Figure S4. Partial dependence plots show predicted printability's dependency on each feature's value. Features were ranked from A to J based on their importance determined by SHAP analysis.
